# Semi-Quantification of Myocardial Uptake of Bone-Seeking Agents in Suspected Cardiac Amyloidosis

**DOI:** 10.3390/jcdd10050184

**Published:** 2023-04-22

**Authors:** Cristina Campi, Chiara Briani, Alessandro Salvalaggio, Chiara Giraudo, Alberto Cipriani, Alessandro Zorzi, Pietro Zucchetta, Roberto Vettor, Diego Cecchin

**Affiliations:** 1 Department of Mathematics, University of Genoa, 16126 Genoa, Italy; campi@dima.unige.it; 2 Department of Neurosciences (DNS), University of Padua, 35128 Padua, Italy; chiara.briani@unipd.it (C.B.); alessandro.salvalaggio@unipd.it (A.S.); 3 Nuclear Medicine Unit, Department of Medicine—DIMED, Padua University Hospital, 35128 Padua, Italy; chiara.giraudo@unipd.it (C.G.); pietro.zucchetta@unipd.it (P.Z.); 4 Department of Cardiac, Thoracic, Vascular Sciences and Public Health, Padua University Hospital, 35128 Padua, Italy; alberto.cipriani@unipd.it (A.C.); alessandro.zorzi@unipd.it (A.Z.); 5 Internal Medicine Unit, Department of Medicine—DIMED, Padua University Hospital, 35128 Padua, Italy; roberto.vettor@unipd.it

**Keywords:** transthyretin, cardiac amyloidosis, bone scintigraphy, bone scan, quantification

## Abstract

Introduction: Bone scintigraphy has emerged as a key tool for non-invasive etiologic diagnosis of transthyretin (ATTR) cardiac amyloidosis (CA). We focused on a new semi-quantification method (on planar imaging) that could complement the qualitative/visual Perugini scoring system, especially when SPET/CT is not available. Material and Methods: We retrospectively/qualitatively evaluated 8674 consecutive, planar 99mTc-biphosphonate scintigraphies (performed for non-cardiac reasons), identifying 68 (0.78%) individuals (mean age 79 ± 7 years, range 62–100 years; female/male ratio 16/52) presenting myocardial uptake. Due to the retrospective nature of the study, no SPET/CT, pathologic or genetic confirmation was obtained. The Perugini scoring system was determined (in patients presenting cardiac uptake) and compared with three newly proposed semi-quantitative indices. We took 349 consecutive bone scintigraphies, qualitatively absent of any cardiac/pulmonary uptake, as “healthy controls” (HC). Results: The heart-to-thigh ratio (RHT) and lung-to-thigh ratio (RLT) indices were significantly higher in patients than in HCs (p ≤ 0.0001). There were statistically significant differences for RHT in HCs vs. patients with qualitative Perugini scores of 1 or >1 (with p ranging from ≤0.001 to ≤0.0001). ROC curves showed that RHT outperformed the other indices and was more accurate in both male and female groups. Furthermore, in the male population, RHT accurately distinguished HCs and patients with scores of 1 (less likely affected by ATTR) from patients with qualitative scores >1 (more likely affected by ATTR) with an AUC of 99% (sensitivity: 95%; specificity: 97%). Conclusion: The proposed semi-quantitative RHT index can accurately/semi-quantitatively distinguish between HCs and subjects probably affected by CA (Perugini scores from 1 to 3), and could be particularly useful when no SPET/CT data are available (such as in retrospective studies and data mining). Furthermore, RHT can semi-quantitatively predict, with very high accuracy, subjects in the male population more likely to be affected by ATTR. The present study, although using a very large sample, is however retrospective, monocentric, and therefore the generalizability of the results should be proved by an accurate external validation. Advances in Knowledge: The proposed heart-to-thigh ratio (RHT) can distinguish healthy controls and subjects that are probably affected by cardiac amyloidosis in a simple and more reproducible way, as compared to standard qualitative/visual evaluation.

## 1. Introduction

Cardiac amyloidosis (CA) is an underdiagnosed cardiomyopathy characterized by diastolic dysfunction, restrictive hemodynamics, and heart failure resulting from the progressive deposition of insoluble amyloid fibrils in the myocardium [1]. On the basis of the molecular structure of the amyloid deposits, it is possible to distinguish three main etiologies of CA, which together account for approximately 98% of clinical cases [2]: primary light chain (AL) amyloidosis, hereditary transthyretin (ATTRv; v = variant) and wild-type transthyretin (ATTRwt) amyloidosis. While the misfolding, accumulation and deposit of immunoglobulin light chains (monoclonal kappa or lambda) lead to AL CA [3], amyloid formation and deposition in transthyretin amyloidosis results from dissociation of the tetrameric transthyretin molecule (due either to a genetic mutation, ATTRv, or ageing in ATTRwt) and the misfolding of the resulting monomers into amyloid fibrils [4].

The gold standard for CA diagnosis and subtyping is endomyocardial biopsy (EMB) and histopathological and immunohistochemical analysis [5], a procedure that has optimal sensitivity and specificity [6,7], but has the disadvantages of high cost, invasiveness, and life-threatening complications in approximately 6% of patients [2]. 

A number of non-invasive diagnostic tools have so far been used in CA, including serum cardiac biomarkers (e.g., troponin T and NT-proBNP) [8], strain echocardiography [9,10], and cardiac magnetic resonance imaging [11]. However, none of these procedures provides an accurate characterization of the amyloidogenic protein, which is fundamental for correct treatment planning [12]. 

Among the nuclear medicine molecular targets, conventional bone-seeking agents (high myocardial uptake is associated with ATTR CA subtypes, hereditary and wild-type), and amyloid PET {high myocardial uptake of 11C-PIB, 18F-Flutemetamol or 18F-Florbetaben has been associated with the AL CA subtype [13] and ATTRV30M amyloidosis [14]} have emerged as key procedures for the non-invasive etiologic diagnosis of cardiac amyloidosis (CA), allowing for the early instigation of therapy.

In the present study, we focused on bone-seeking agents because these radiopharmaceuticals have been more widely used in nuclear medicine to diagnose CA, and also because cardiac uptake may be incidentally found when a bone scan is performed for oncological/rheumatological indications.

The possibility of distinguishing different CA subtypes was suggested by Perugini et al. on the basis of an initial study, with 15 patients with ATTR and 10 patients with AL CA who underwent 99mTc-DPD scans. Radiotracer uptake was reported in all ATTR cases and in none of the AL patients [15]. Although a second study with a higher number of patients (45 ATTR, 34 AL, and 15 control subjects) identified mild 99mTc-DPD uptake in about one third of AL cases [16], a very high uptake of 99mTc-DPD is usually associated with the ATTR CA subtype [17].

It has been recently demonstrated [18] that the death rate and heart failure are higher in patients with a Perugini score ≥ 2 with respect to controls and patients with a Perugini score of 1. Due to the frequent need for specific, objective cut-off values both for diagnosis and for therapeutic management and follow-up, a semi-quantitative approach may be more useful [18] than a purely qualitative approach in clinical practice, especially to disambiguate qualitative doubtful cases.

Therefore, in this study we focused on a new semi-quantification method using bone-seeking agents (and planar whole-body acquisition) with the aim of disambiguating qualitative doubtful cases (in view of new therapeutic possibilities) and testing the developed simple semiquantitative method against the validated, but qualitative, Perugini score.

To do so, we derived a ratio between the mean value uptake of the heart and the mean value of the uptake by the medial portion of both thighs, because the chosen reference area should ideally be free from the disease of interest (a qualitative visual assessment excluded the lung, contralateral mediastinum, bone and muscles being sites of significant uptake in the pathologic population).

It should be emphasized, however that being a retrospective study (on bone scans acquired for non-cardiac reasons), although we succeeded in separating HC from patients (presenting a positive Perugini score) we were not able to retrieve all clinical data, underlying mutations or phenotypes to confirm the true biological diagnosis.

## 2. Materials and Methods

A total of 8674 consecutive 99mTc-biphosphonate (HMDP or DPD) scintigraphies, performed for oncological staging or rheumatologic diseases, were retrospectively evaluated in order to identify patients showing significant cardiac uptake (none was acquired specifically because of a suspected CA, because this is still an off-label use—at least, in our country) at the Nuclear Medicine Unit between January 2012 and December 2016 (this time period was chosen to have mixed HMDP and DPD cases, because DPD was used in our center from 2012 to mid-2014 and HMPD from mid-2014 to 2016). Patients aged < 18 years were excluded from the study. The local ethical committee approved the retrospective study with the reference number 24n/AO/20.

All bone scans were performed 2.5 to 4 h after intravenous administration of 600–800 MBq of 99mTc-biphosphonates, depending on the patient’s weight and age, using either a dual-headed gamma camera equipped with low-energy, high-resolution (HR) parallel hole collimators (InfiniaTM, GE Healthcare, Waukesha, WI, USA) or a triple-headed gamma camera with low-energy, ultra-high resolution (UHR) parallel hole collimators (IrixTM, Philips Medical System, formerly Marconi/Picker, Holland). Delayed (2.5–4 h) whole body images in anterior and posterior views were acquired using a predefined time frame (20–25 min at a scanning speed of 15–18 cm/min) and a matrix size of 1024 × 256. Image statistics, resolution and signal-to-noise ratio, for the purpose of the present study, are comparable between used systems. 

When myocardial uptake was observed qualitatively on planar images, the data were processed as follows:

Qualitative analysis: each patient was classified by a nuclear medicine physician with 18 years of experience, and by two in-training nuclear medicine physicians with 3 years of experience, who jointly reached a consensus using the standard qualitative visual score described by Perugini et al. [15] (SPET data were available for only a small minority of patients and were excluded from the analysis):

- score 0, no cardiac uptake and normal bone uptake
- score 1, mild cardiac uptake that is less than bone uptake
- score 2, moderate cardiac uptake accompanied by attenuated bone uptake
- score 3, strong cardiac uptake with mild/absent bone uptake

Furthermore, we gave a ‘doubtful’ classification to a number of cases where cardiac uptake did not properly fit the Perugini qualitative score, i.e., uptake was too mild to be classified as 1, but was not completely absent. In these cases, a fourth nuclear medicine physician with 25 years of experience confirmed the “doubtful” classification.

**Figure 1:**
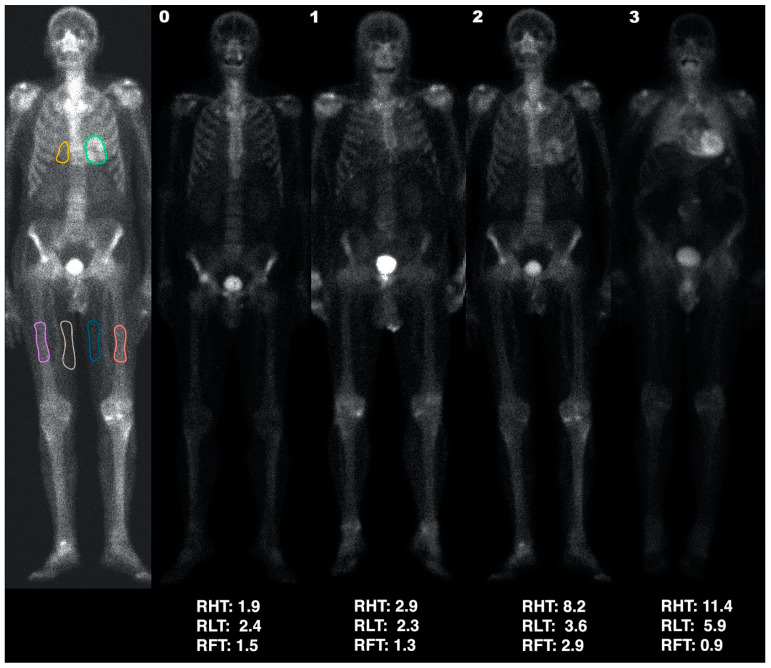
First column: ROIs (heart: green; lung: yellow; thighs: blue and white; bone: pink and orange) used to calculate the RHT, RLT, and RFT indices (presented under each patient). Examples are of an HC (score 0; second column) and patients with scores of 1, 2, and 3 (third, fourth and fifth columns, respectively).

Semi-quantitative analysis: cardiac uptake was then evaluated using a semi-quantitative method, which covered:

- Heart uptake: regions of interest (ROIs) were manually drawn on the anterior view as accurately as possible around the heart uptake, excluding ribs and sternum, and on the medial portion of the soft tissues (therefore mainly corresponding to adipose tissue) of both the mid-thighs (Figure 1), excluding vessel uptake. The heart-to-thigh ratio (RHT) was then computed as (heart average counts—mid-thigh average counts)/mid-thigh average counts.
- Pulmonary uptake: since most patients with bone tracer cardiac uptake also showed a particularly increased pulmonary uptake, the lungs were also semi-quantitatively assessed. A ROI in the right lung, including part of the mediastinum and excluding ribs (at least in the anterior view) and sternum, was drawn as accurately as possible. The semi-quantitative lung to thigh ratio (RLT) was then computed as (pulmonary average counts—mid-thigh average counts)/mid-thigh average counts.
- Bone uptake: since patients with intense cardiac uptake often showed an apparent reduction in bone uptake, we also defined ROIs on both femoral diaphyses (Figure 1) of each patient with cardiac uptake. The semi-quantitative femur-to-thigh ratio (RFT) was then computed as (femoral average counts—mid-thigh average counts)/mid-thigh average counts.

Dataset of normal subjects: to obtain a consistent normal reference database (referred to as “healthy controls” or HC, although biological/genetic confirmation is lacking) from which to determine cut-offs, of the 8677 consecutive bone scintigraphies examined (excluding positive and doubtful cases), we selected 349 consecutive scans where no cardiac/pulmonary uptake was qualitatively observed and where the subjects were aged > 50 years. RHT, RLT, and RFT were calculated for each HC. The relatively high number of HC individuals compared with the patient population was due to the need to split the HC cohort by gender (males vs. females), and to verify whether the reference values varied with age/sex. For this purpose, we grouped HCs as follows: females aged 50–64 years (healthy young females: HYF), females > 65 years (healthy elderly females: HEF), males aged from 50–64 years (healthy young males: HYM), males > 65 years (healthy elderly males: HEM).

### Statistical Analysis

The population was divided into three groups: healthy controls (HC), patients, and doubtful cases.

An unpaired *t*-test was used to compare the three ratios—RHT, RHT, and RFT—among the three groups. Statistical significance was set at <0.05.

Receiver operating characteristic (ROC) curves were constructed and the Youden’s index calculated to determine the cut-off value that best discriminated between the HC and patient groups. The cut-offs were then used to classify the doubtful group. 

We also determined the cut-off values for discriminating the HCs and patients with a score of 1 versus the patients with scores of 2 and 3. 

Statistical analyses were performed using the R software and the pROC package [19]. 

## 3. Results

The averages ± standard deviations of the demographic data and ratios values are summarized in Table 1. The HC population comprised 349 subjects with a mean age of 66 ± 10 years (range 50–90 years; 166 females, 183 males). 

The patient population comprised 68 individuals with a mean age of 79 ± 7 years (range 62–100 years; 16 females, 52 males), and there were 27 patients classified as doubtful (mean age: 70 ± 10 years, range: 49–86 years; 11 females, 16 males). 

Of the patient population (n = 68), 43 (63%) had a Perugini score of 1, 18 (27%) a score of 2, and 7 (10%) a score of 3.

### 3.1. Analysis

#### 3.1.1. HC Analysis

Analysis by the Kruskal–Wallis test of the variability in the ratios according to age and sex in the HC cohort showed no significant differences between the two male HC classes (HYM vs. HEM) in any of the three ratios (*p* > 0.80), while significant differences between the two female HC classes (HYF vs. HEF) were found in RHT (*p* < 0.00001), and in RLT and RFT (*p* < 0.01). Significant differences were also found between healthy males and females in all three ratios (HYF vs. HYM: *p* < 0.00001; HEF vs. HEM: *p* < 0.01). Therefore, in order to accurately match the average ages of the female populations (pathologic and HC), we excluded female HCs under 65. The new HC population comprised 89 females and 183 males, comparable by age and sex to the selected subjects presenting cardiac uptake.

To determine whether the chosen reference area (mean of counts in ROIs positioned on both thighs) was responsible for the observed differences in gender and age, the mean values of the reference areas in HYF, HEF, HYM, and HEM were calculated and compared. A Kruskal–Wallis test revealed statistically significant differences in the reference areas between HYF and HEF (*p* < 0.001), between HYF and HYM (*p* < 0.01), and between HYF and HEM (*p* < 0.0001), but there were no differences between HYM and HEM (*p* = 0.12). 

#### 3.1.2. Overall Analysis

**Figure 2:**
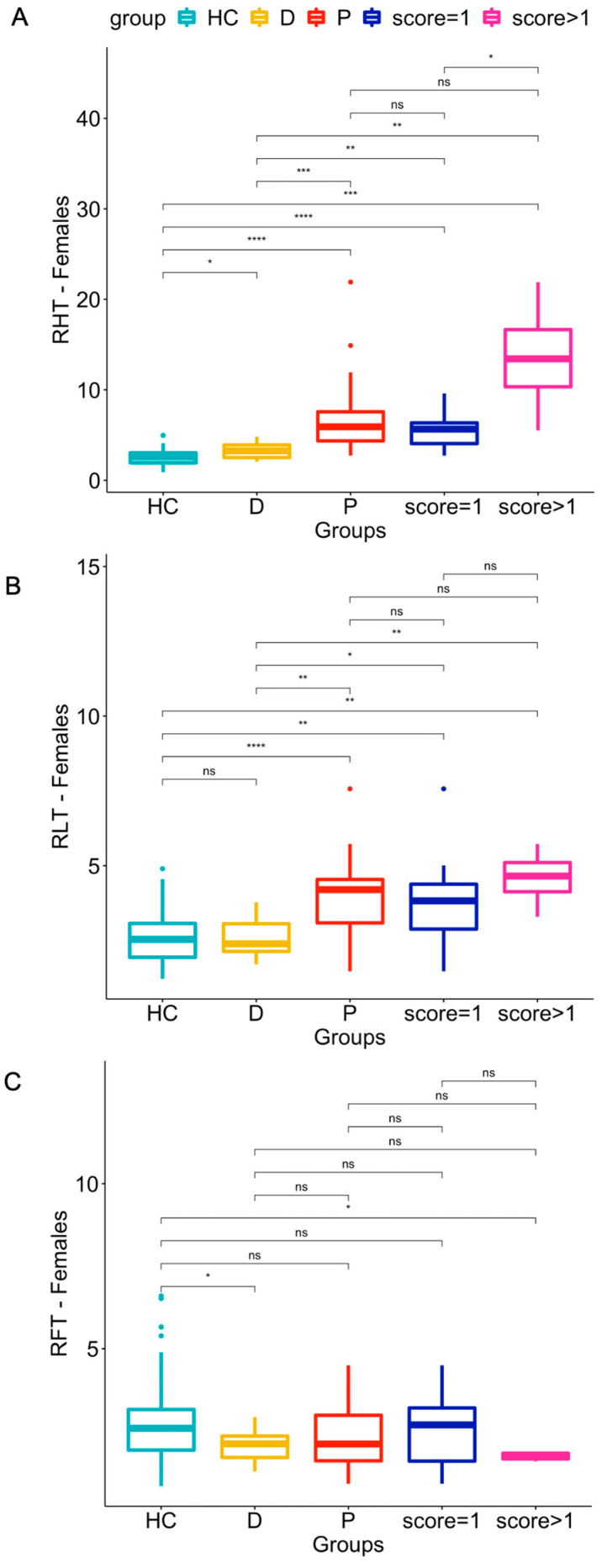
Distribution of RHT (panel **A**), RLT (panel **B**), and RFT (panel **C**), values for healthy controls (HC), doubtful cases (D), patients (P), patients with score = 1, and patients with scores >1 across the female population. ns: *p* > 0.05; * *p* ≤ 0.05; ** *p* ≤ 0.01; *** *p* ≤ 0.001; **** *p* ≤ 0.0001.

**Figure 3:**
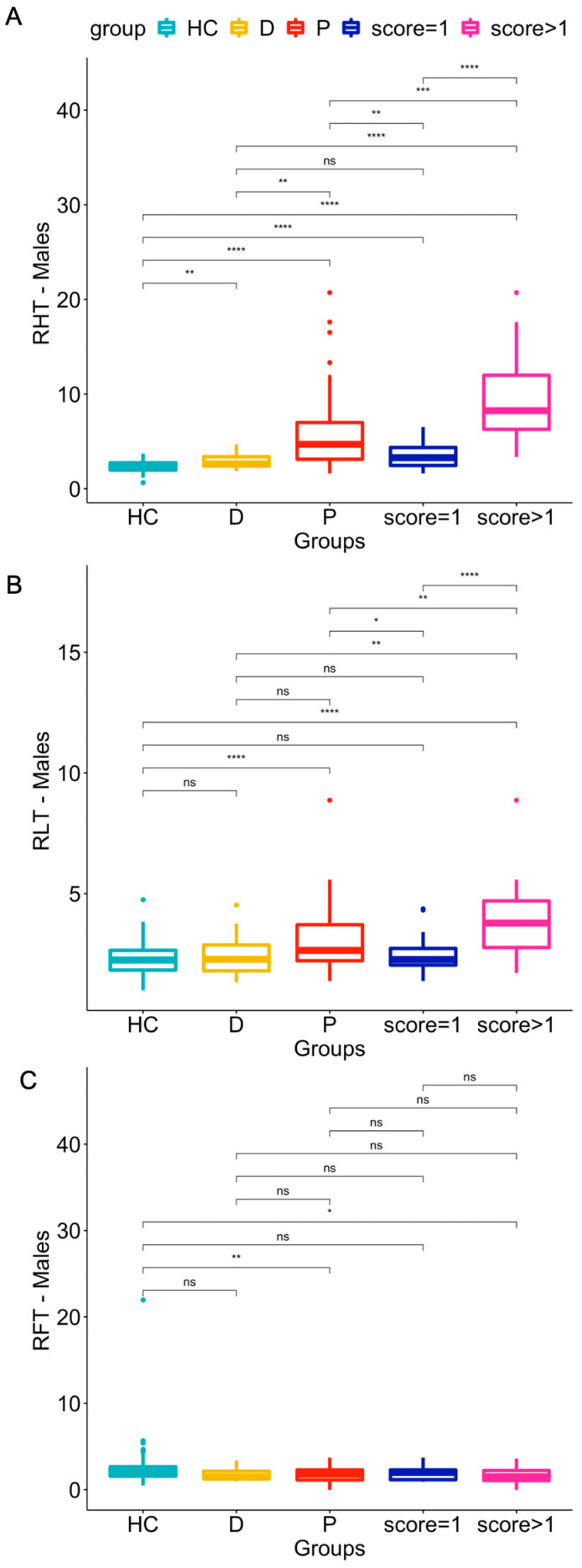
Distribution of RHT (panel **A**), RLT (panel **B**), and RFT (panel **C**), values for healthy controls (HC), doubtful cases (D), patients (P), patients with score = 1, and patients with scores >1 across the male population. ns: *p* > 0.05; * *p* ≤ 0.05; ** *p* ≤ 0.01; *** *p* ≤ 0.001; **** *p* ≤ 0.0001.

The results of the statistical comparison by Kruskal–Wallis test of the three computed ratios (RHT, RHT, and RFT) in male vs. female populations (HC, patients and doubtful cases) are shown in Figure 2 and Figure 3.

Regarding the Female Group:
  - RHT and RLT were significantly higher in patients than in HCs (*p*-values ≤ 0.0001) (see panel A and B of Figure 2). Both indices also differed significantly in patients with qualitative scores of 1 or >1 versus HCs (*p* ranging from ≤0.01 to ≤0.0001, with the lower levels of significance for RLT compared to RHT).
  - Only two statistically significant (*p* ≤ 0.05) differences in RFT were found: between HC and doubtful patients, and between HC and patients with scores > 1 (see Figure 2, panel C).
  - Doubtful patients differed significantly (*p* ≤ 0.05) from HCs in the RHT and RFT indices but not in RLT.
Regarding the male group:
  - The RHT and RLT indices in patients were significantly higher than in HCs (*p*-values ≤ 0.0001) (see panel A and B of Figure 3). RHT was also significantly higher in patients with qualitative scores of 1 or >1 (*p* ≤ 0.0001) than in HCs.
  - Only two statistically significant differences in RFT were found: between HCs and patients (visual scores from 1 to 3; *p* ≤ 0.01), and between HCs and patients with scores > 1 (*p* ≤ 0.05) (see Figure 3, panel C).
  - Doubtful patients differed significantly (*p* ≤ 0.05) from HCs in the RHT index but not in RLT or RFT.

#### 3.1.3. Cut-offs by ROC Curves

ROC curves and the corresponding values (sensitivity, specificity, cut-offs and AUC) for the three ratios (RHT, RHT, and RFT) in the HC and patient groups were computed separately for female and male populations. The results are shown in Table 2 (females) and Table 3 (males). The area under the curve (AUC) of RHT in terms of significantly detecting cardiac uptake (scored visually from 1 to 3) of the bone tracer was 0.96 (95% CI 0.90–1.00) in females, and 0.87 (95% CI 0.80–0.94) in males. The best cut-off value of RHT for females was 3.260, which provided a sensitivity of 94%, and a specificity of 87%. For males, the best cut-off value of RHT was 2.965, which provided a sensitivity of 81% and a specificity of 88%. Therefore, derived cut-off values were used to re-classify female (Table 4) and male (Table 5) individuals that were previously qualitatively classified as doubtful. We were thus able to reclassify as patients 6/11 (54.5%) females and 5/16 (31.25%) males using RHT, 2/11 (18%) females and 2/16 (12.5%) males using RLT, and 2/11 (18%) females and 6/16 (38%) males using RFT.

To ascertain whether the proposed indices were also able to reliably distinguish HCs and patients with a score of 1 (known, from literature, to be less likely affected by ATTR) from patients with qualitative scores > 1 (known, from literature, to be more likely affected by ATTR), we grouped the male population on the basis of their visual Perugini (P) scores (HC + P1 vs. P2 + P3). We were not able to do the same for the female population due to the small sample size. We used the cut-off values (shown in Table 6 and Table 7) and were able to re-classify as probable ATTR 1/16 (6.25%) doubtful male subjects using RHT, and 2/16 (12.5%) using RLT.

## 4. Discussion

In the present study, new semi-quantitative methods for the assessment of myocardial uptake of bone-seeking agents were retrospectively tested in a large cohort of patients undergoing bone scintigraphy for different, non-cardiac, clinical reasons. 

It should be underlined that patient population and HC were roughly scanned 50% with DPD and 50% with HMDP. A separate analysis of the two tracers goes beyond the scope of the paper but, based on the available literature, we do not expect significant differences between the two tracers. Although the exact binding mechanism remains a matter of debate, in fact, two bone-seeking agents have been shown to be comparably effective in localizing cardiac amyloid deposits: 3,3diphosphono-1,2-propanodicarboxylic acid (99mTc-DPD), and more recently 99mTc-HMDP (hydroxymethylene diphosphonate) [20,21,22]. Few studies have investigated the usefulness of 99mTc-HMDP for diagnosing ATTR CA. Cappelli et al. performed 99mTc-HMDP scans on 65 biopsy-proven CA patients (26 AL, 39 ATTR) and reported no amyloid uptake in 92% of AL CA patients, and moderate/strong uptake in 93% of ATTR CA patients [20]. Abulizi et al. performed 99mTc-DPD and 99mTc-HMDP scans (of six biopsy-proven ATTR CA patients) and reported similar heart/mediastinum ratio values for both tracers [22]. More recently, Musumeci et al. showed [23] both 99mTc-DPD and 99mHMDP to have both low levels of accuracy with regard to Phe64Leu mutation-related transthyretin CA.

Due to the retrospective nature of the study, only planar bone scans have been used. Qualitative visual assessment of myocardial uptake on planar images, as proposed by Perugini et al., is the most widely-adopted and easy method to evaluate CA [15]. However, as it is based on a discrete 4-point scale (rather than on a continuous semi-quantification index), it could be prone to subjective errors and misclassifications, especially when dealing with doubtful cases. A more recent study by Hutt et al. demonstrated cardiac uptake on SPET/CT scans in seven CA patients (5 AL with median Grade 1 intensity, and 2 ATTRv) that would otherwise have been missed on planar image evaluation by two experienced nuclear medicine specialists [24]. These authors therefore proposed revising the Perugini visual scoring system by taking into account SPET/CT and soft-tissue uptake. It should be noted, however, that in several countries the use of bone seeking agent for the confirmation of CA is still considered off-label, and therefore heart uptake (although rare) could be more frequently found on clinical whole body planar images (performed for oncological or rheumatological reasons) rather than on dedicated cardiac SPET acquisitions. Furthermore, sometimes SPET/CT acquisitions could not be achieved (demented, claustrophobic, etc.).

The main result is that the proposed RHT semi-quantitative index was able to distinguish between healthy controls and patients with significant cardiac uptake, i.e., with Perugini visual scores from 1 to 3, with an AUC of 96% (sensitivity: 94%, specificity: 87%) in the female population, and an AUC of 87% (sensitivity: 81%, specificity: 88%) in the male population. Using RHT, we were able to re-classify as positive a number of subjects—6/11 (54.5%) females, and 5/16 (31.2%) males—that were previously qualitatively classified as doubtful. The proposed RHT metric may, therefore, help improve qualitative interpretation, especially in doubtful cases, and could be used in longitudinal evaluations where qualitative analysis is known to be limited.

More interestingly, in the male population, RHT was also able to reliably distinguish HCs and patients with a score of 1 (known to be less likely affected by ATTR) from patients with qualitative scores >1 (known to be more likely affected by ATTR) with an AUC of 99% (sensitivity: 95%, specificity: 97%), allowing us to qualitatively reclassify doubtful patients as probable ATTR. This is crucial, given the new treatments approved for ATTR [1], as the more objective semi-quantitative criteria may improve disease management by prompting genetic testing, leading to early initiation of appropriate therapeutic treatments.

It should be noted, in fact, as recently proposed [25], that the accurate identification (possible with the proposed semi-quantitative ratios) of a patient with a Perugini score of 1, without monoclonal gammopathy (MG), is strongly suggestive of early ATTR-type and could prompt urgent histologic corroboration. We are strongly convinced that the accurate identification of patients with a semiquantitative initial positivity (the equivalent to Perugini score 1) could be one of the main clinical applications of the proposed method. A number of authors [26,27] have emphasized the importance of an early diagnosis to identify who may be eligible for newly available treatments to ameliorate the prognosis of this devastating disease.

As for the majority of ratios used in nuclear medicine, the chosen reference area should ideally be free from the disease of interest (in our case, CA). In the present paper, the mean value of the uptake by the medial portion of both thighs (excluding vessel uptake, and therefore mainly referrable to adipose tissue) was used as the reference area, as a qualitative visual assessment found the lung (or contralateral mediastinum frequently used in the heart-to-controlateral lung ratio), bone and muscles to be sites of significant uptake in the pathologic population. A significant difference in the mean values of the reference area between “young” and “old” females and between genders was observed, which were lower in the young female group compared with the other groups. This may be due to the different lipidic contents (in the medial thigh soft tissue) of young females compared with older females and males, probably because of the modulatory role of sex steroids. The HEF group (i.e., excluding the younger female subjects) was therefore taken as the normal population, because all of the female pathologic subjects (except 1 doubtful patient) could be considered age matched with this group. No statistically significant age-related differences in the reference area were observed in the male population.

Although the proposed RLT metric differed significantly between HCs and subjects with significant cardiac uptake (panel B in Figure 2 and Figure 3 showing overlap), it performed worse than RHT, presenting an AUC of 81% (sensitivity: 88%, specificity: 66%) in the female population, and an AUC of 67% (sensitivity: 33%, specificity: 95%) in the male population. Despite its likely unsuitability for clinical use in diagnosing CA, the RLT index and the comparisons with HCs confirm the visual impression of increased uptake by the lungs of patients with CA. It therefore seems inappropriate to use a heart-to-contralateral side (as the reference area) ratio as an index for CA.

Concerning the RFT metric, although several mildly significant differences were found in HCs vs. patients (visual scores from 1 to 3), doubtful subjects or patients with scores >1 (panel C in Figure 2 and Figure 3 showing wide overlap) in the ROC curve analysis revealed too low a sensitivity (50% for the female group, 38% for the male group) for RFT to be considered a useful index for distinguishing HC from CA subjects. A similarly low sensitivity (57%; Table 6) was also found in the male population when HCs and subjects with a visual score of 1 were grouped against subjects with visual scores > 1. Nevertheless, we consider this analysis to be useful since it shows that “attenuated bone uptake” or “mild/absent bone uptake”, as indicated by the Perugini scores refer mainly to how the images are normalized rather than to any real reduction in bone uptake caused by CA.

### Limitations

The bias of having mixed gamma cameras/collimators and protocols that were not identical (although very similar) is a consequence of the retrospective nature of the study. Image statistics, resolution, and signal-to-noise ratio, for the purpose of the present study, are comparable between used systems. Slight differences among systems, on the other hand, may simulate the heterogeneity of the real-world diagnostic setting. This bias may have also been partially mitigated by the fact that ratios (instead of absolute values) were used to calculate the indices.

There is growing evidence that a SPET/CT rather than a planar acquisition would be desirable (also for semi-quantification) in CA, but due to the retrospective nature of the study it was not possible to obtain such data. It should be noted, however, that SPET/CT is not always available or obtainable (demented, claustrophobic). We believe therefore that a simple and robust semi-quantification method based on planar images could be a reliable simplification that could, at least, substitute the qualitative/visual assessment (prone to subjective errors, as demonstrated), especially when SPET/CT is not available.

The Perugini score, commonly accepted both in research and in a clinical setting, was used in the present paper as a gold standard to evaluate the performances of semi-quantitative methods, because no genetic testing, cardiac MR, strain echocardiography or pathology data was available for the retrospectively identified population. Furthermore, it will not be possible to perform any further analysis because the majority of oncologic patients have died. Although no pathologic or genetic confirmation was obtained in this study, an accurate retrospective examination of their medical records revealed that 12/25 patients with visual scores of 2 or 3 presented with heart failure and concentric hypertrophy of the left ventricle, supporting the hypothesis that this was a real ATTR population. We have no follow-up data for the remaining 13/25.

The present study, although using a very large sample, is retrospective and monocentric; therefore, the generalizability of results should be proved by an accurate external validation.

Further studies are also desirable to determine whether the proposed planar semi-quantitative RHT index is robust compared to SPET/CT data and pathology, if it is able to depict variations in heart uptake over time, and therefore could be used to monitor disease progression.

## 5. Conclusions

The proposed semi-quantitative RHT index is able to accurately distinguish HCs from subjects with significant cardiac uptake of bone-seeking agents (with Perugini scores from 1 to 3) in both females and males, even without SPET/CT availability. Furthermore, in the male population, RHT distinguished HCs and patients with a score of 1 (known to be less likely affected by ATTR) from patients with qualitative scores >1 (known to be more likely affected by ATTR) with a high level of accuracy (AUC of 99%—sensitivity: 95%; specificity: 97%).

## Figures and Tables

**Table 1:** Demographic data and ratio values for the whole population grouped by males and females. HC: healthy controls; D: doubtful patients; P score = 1-2-3: groups of patients with Perugini scores of 1-2-3, respectively; P-total: all patients with scores of 1, 2, and 3.

	HC	D	P Score = 1	P Score = 2	P Score = 3	P-Total
FEMALES:						
Number	89	11	12	3	1	16
Age (years)	73 ± 6	71 ± 8	78 ± 7	81 ± 5	83	79 ± 6
Age range (years)	65–90	49–79	66–88	77–86	83	66–88
RHT	2.52 ± 0.75	3.30 ± 0.93	5.39 ± 1.92	10.79 ± 4.79	21.91	7.43 ± 5.03
RLT	2.58 ± 0.85	2.62 ± 0.71	3.83 ± 1.56	4.20 ± 0.83	5.73	4.02 ± 1.45
RFT	2.74 ± 1.17	2.13 ± 0.61	2.60 ± 1.12	1.70 ± 0.11	1.88	2.39 ± 1.03
MALES:						
Number	183	16	31	15	6	52
Age (years)	68 ± 9	68 ± 11	78 ± 8	80 ± 6	82 ± 5	79 ± 7
Age range (years)	50–86	50–86	62–100	69–86	72–89	62–100
RHT	2.28 ± 0.57	2.80 ± 0.83	3.55 ± 1.39	8.39 ± 3.55	12.34 ± 6.14	5.96 ± 4.25
RLT	2.26 ± 0.60	2.35 ± 0.92	2.44 ± 0.68	3.53 ± 0.94	4.92 ± 2.20	3.04 ± 1.33
RFT	2.31 ± 1.77	1.72 ± 0.70	1.87 ± 0.73	1.91 ± 1.02	1.35 ± 1.12	1.82 ± 0.84

**Table 2:** Sensitivity, specificity, cut-offs, and AUC values (95% CI) for the three ratios in the female population.

	Females (n = 105)			
	HC (n = 89), P (n = 16)			
	Sensitivity (95% CI)	Specificity (95% CI)	Cut-Off	AUC (95% CI)
RHT	0.94 (0.81; 1.00)	0.87 (0.79; 0.93)	≥3.260	0.96 (0.90; 1.00)
RLT	0.88 (0.69; 1.00)	0.66 (0.57; 0.76)	≥2.825	0.81 (0.68; 0.95)
RFT	0.50 (0.25; 0.75)	0.76 (0.67; 0.76)	<1.890	0.59 (0.43; 0.76)

**Table 3:** Sensitivity, specificity, cut-offs and AUC values (95% CI) for the three ratios in the male population.

	Males (n = 235)			
	HC (n = 183), P (n = 52)			
	Sensitivity (95% CI)	Specificity (95% CI)	Cut-Off	AUC (95% CI)
RHT	0.81 (0.69; 0.90)	0.88 (0.83; 0.93)	>2.965	0.87 (0.80; 0.94)
RLT	0.33 (0.19; 0.46)	0.95 (0.92; 0.98)	>3.290	0.67 (0.59; 0.76)
RFT	0.38 (0.25; 0.52)	0.86 (0.81; 0.91)	<1.305	0.62 (0.53; 0.71)

**Table 4:** Re-classification of the qualitatively doubtful female patients on the basis of the semi-quantitative cut-off values.

Doubtful	Females (n = 11)
	Cut-Off
RHT	n = 6 (54.54%) ≥ 3.260
RLT	n = 2 (18.18%) ≥ 2.825
RFT	n = 2 (18.18%) < 1.890

**Table 5:** Re-classification of qualitatively doubtful male patients on the basis of the semi-quantitative cut-off values.

Doubtful	Males (n = 16)
	Cut-Off
RHT	n = 5 (31.25%) > 2.965
RLT	n = 2 (12.5%) > 3.290
RFT	n = 6 (37.5%) < 1.305

**Table 6:** Sensitivity, specificity, cut-offs and AUC values (95% CI) for the three ratios in the male sub-group computed on the basis of patients with a qualitative score of 1 + HC versus patients with qualitative scores >1 (2 and 3).

	Males (n = 235)			
	HC + Score = 1 (n = 214) vs. Score >1 (n = 21)			
	Sensitivity (95% CI)	Specificity (95% CI)	Cut-Off	AUC (95% CI)
RHT	0.95 (0.86; 1.00)	0.97 (0.95; 0.99)	≥4.69	0.99 (0.98; 1.00)
RLT	0.66 (0.47; 0.86)	0.94 (0.91; 0.97)	≥3.29	0.86 (0.76; 0.96)
RFT	0.57 (0.38; 0.76)	0.75 (0.69; 0.81)	<1.515	0.63 (0.48; 0.78)

**Table 7:** Re-classification of qualitatively doubtful male patients using the semi-quantitative cut-off values, computed on the basis of patients with a qualitative score of 1 versus patients with scores > 1 (2 and 3).

Doubtful	Males (n = 16)
	Cut-Off
RHT	n = 1 (6.25%) ≥ 4.69
RLT	n = 2 (12.5%) ≥ 3.29
RFT	n = 9 (56.25%) < 1.515

## Data Availability

Data are unavailable due to privacy and ethical restrictions.
